# Erector Spinae Plane Block for Perioperative Analgesia after Percutaneous Nephrolithotomy

**DOI:** 10.3390/ijerph18073625

**Published:** 2021-03-31

**Authors:** Piotr Bryniarski, Szymon Bialka, Michal Kepinski, Anna Szelka-Urbanczyk, Andrzej Paradysz, Hanna Misiolek

**Affiliations:** 1 Department of Urology in Zabrze, Faculty of Medical Sciences in Zabrze, Medical University of Silesia in Katowice, 40-055 Katowice, Poland; kepinskimm@gmail.com (M.K.); parady@poczta.onet.pl (A.P.); 2 Department of Anaesthesiology, Intensive Care and Emergency Medicine, Faculty of Medical Sciences in Zabrze, Medical University of Silesia in Katowice, 40-055 Katowice, Poland; szymon.bialka@gmail.com (S.B.); ania.szelka@gmail.com (A.S.-U.); hanna.misiolek@gmail.com (H.M.)

**Keywords:** erector spinae plane block, percutaneous nephrolithotomy, urolithiasis

## Abstract

Erector spinae plane block was recently introduced as an alternative to postoperative analgesia in surgical procedures including thoracoscopies and mastectomies. There are no clinical trials regarding erector spinae plane block in percutaneous nephrolithotomy. The aim of our study was to test the efficacy and safety of erector spinae plane block after percutaneous nephrolithotomy. We analyzed 68 patients, 34 of whom received erector spinae plane block. The average visual analogue scale score 24 h postoperatively was the primary endpoint. The secondary endpoints were nalbuphine consumption and the need for rescue analgesia. Safety measures included the mean arterial pressure, Ramsey scale score, and rate of nausea and vomiting. The visual analogue scale, blood pressure, and Ramsey scale were assessed simultaneously at 1, 2, 4, 6, 12, and 24 h postoperatively. The average visual analogue scale was 2.9 and 3 (*p* = 0.65) in groups 1 (experimental) and 2 (control), respectively. The visual analogue scale after 1 h postoperatively was significantly lower in the erector spinae plane block group (2.3 vs. 3.3; *p* = 0.01). The average nalbuphine consumption was the same in both groups (46 mL vs. 47.2 mL, *p* = 0.69). The need for rescue analgesia was insignificantly different in both groups (group 1, 29.4; group 2, 26.4%; *p* = 1). The mean arterial pressure was similar in both groups postoperatively (91.8 vs. 92.5 mmHg; *p* = 0.63). The rate of nausea and vomiting was insignificantly different between the groups (group 1, 17.6%; group 2, 14.7%; *p* = 1). The median Ramsey scale in all the measurements was two. Erector spinae plane block is an effective pain treatment after percutaneous nephrolithotomy but only for a very short postoperative period.

## 1. Introduction

Erector spinae plane block (ESPB) was recently introduced as an alternative to postoperative analgesia in many surgeries including ventral hernia, thoracoscopy and thoracic vertebra surgery, cholecystectomy, and mastectomy [1,2,3,4,5]. It lowers the opioid consumption and thus decreases the rate of its side effects. In ESPB, local anesthetic is reported to be administered into the interfascial plane between the transverse process of the vertebra and the erector spinae muscles, spreading to multiple paravertebral spaces [6]. Case reports have found that ESPB affects both the ventral and dorsal rami, leading to blockage of both visceral and somatic pain [7,8].

Percutaneous nephrolithotomy (PCNL) is the standard urologic procedure used to disintegrate and remove large kidney stones. The procedure is composed of four main steps. Firstly, under ultrasonographic and fluoroscopic guidance, the desired calyx is punctured. A wire is inserted through the needle and is usually positioned in the ureter. The needle may be withdrawn while the wire stays in place. Secondly, dilators are inserted over the wire coaxially up to 30 Fr. Over the 30 Fr dilator, the sheath is inserted to the desired calyx and the dilator may be withdrawn. Thirdly, through the sheath, the nephroscope is inserted, the stone is located, and with ultrasonic or pneumatic lithotripter, the stone is disintegrated. Fourthly, after completion of surgery, the nephrostomy tube is inserted through the tract to drain the kidney. Usually, the nephrostomy is removed on the second postoperative day. PCNL is highly effective procedure; however, it is accompanied by significant postoperative pain and occasionally high blood loss.

There are no clinical trials regarding ESPB in PCNL. The aim of our study was to test the effectiveness and safety of ESPB after PCNL.

## 2. Materials and Methods

### 2.1. Study Design

The study was designed as a randomized controlled trial. A draft of the study was approved by ethics committee of the Medical University of Silesia in 2018 under the number KNW/0022/KB1/70/I/18. Following approval, the study was prospectively registered in the Australian and New Zealand Clinical Trial Registry under the number ACTRN12619000044123.

### 2.2. Sample Size Estimation

The sample size was calculated by choosing a difference of 1 point in the average visual analogue scale (VAS) score as the minimum expected difference between the groups. Setting α = 0.05, assuming a standard deviation of 1.5 points, and investigating 34 subjects for each group, a significant difference could be detected with a power of 85% (one-sided hypothesis). We expected a maximal dropout rate after PCNL of 10% (due to significant bleeding during operation or the need for additional access to the kidney or residual stone).

### 2.3. Inclusion Criteria

The inclusion criteria were:

(a) Patients who provided informed consent for participation in the study;
(b) Age 18–70 years;
(c) American Society of Anesthesiology (ASA) physical status between I and III;
(d) Patients with a body mass index (BMI) < 35;
(e) Patients with kidney stones over 2 cm in diameter, patients with kidney stones 1–2 cm who wished to have PCNL instead of retrograde intrarenal surgery or shockwave lithotripsy (SWL), or patients with kidney stones 1–2 cm with contraindications for SWL;
(f) Single-access PCNL.

### 2.4. Exclusion Criteria

The exclusion criteria were:

(a) Significant residual stones (>1 cm in diameter) after surgery and the need for a second look;
(b) Bleeding diathesis;
(c) A solitary kidney;
(d) A dermal infection in the injection site;
(e) Contrast and drug allergy;
(f) Routine antidepressant, corticosteroid, pain medication, or anticonvulsant usage;
(g) Massive bleeding during operation that required the premature termination of the procedure and clamping the nephrostomy tube;
(h) The need to apply other pain medications or neuroleptics postoperatively;
(i) A mental state preventing the effective use of an intravenous patient-controlled analgesia (PCA) device.

### 2.5. Measures

#### 2.5.1. Primary Endpoint

The primary endpoint was average VAS (cm) from 6 measurements after surgery (assessed 1, 2, 4, 6, 12, and 24 h postoperatively). Patients were asked to indicate the point on the 100 mm line as the equivalent of pain they perceived. The outcome was measured with a ruler. All six measurements were averaged. The outcome is presented in centimeters.

#### 2.5.2. Secondary Endpoints

The secondary endpoints were:

(a) Nalbuphine consumption (mL) in patient-controlled anesthesia (PCA) during the 24 h postoperative period;
(b) Need for rescue analgesia during the 24 h postoperative period;
(c) Mean arterial pressure (MAP = 1/3 systolic blood pressure + 2/3 diastolic blood pressure, mmHg) assessed 1, 2, 4, 6, 12, and 24 h postoperatively;
(d) Ramsey sedation scale 1, 2, 4, 6, 12, and 24 h postoperatively;
(e) The rate of nausea and vomiting in the 24 h postoperative period.

### 2.6. Interventions

The patients were randomly assigned to receive either standard general anesthesia with subsequent pain management based on intravenous PCA-delivered opioid (Control group, or preoperative ESPB and standard general anesthesia with a PCA pump in the postoperative period (ESPB group). Random assignment was ensured using a sequence generated by a free resource for researchers (www.randomizer.org). Allocation concealment was ensured as the numbers were placed into sealed opaque envelopes and randomly chosen by the anesthetist scheduled to administer anesthesia.

For anesthesia, standard monitoring procedures including pulse oximetry, electrocardiography, and noninvasive arterial pressure measurements were performed prior to anesthesia. Baseline heart rates, systolic and diastolic blood pressures, and mean arterial pressures were recorded. General anesthesia was induced using a bolus of 2 mg/kg propofol intravenously, 100 µg fentanyl intravenously, and 0.6–0.8 mg/kg rocuronium bromide intravenously. We used 0.8–1.2 MAC sevoflurane via endotracheal tube and 0.08 µg/kg/min remifentanil intravenously for anesthesia maintenance. Remifentanil dosage was adjusted according to hemodynamic parameters, up to 2 µg/kg/min. The dose of remifentanil was increased when patient heart rate (HR) or mean blood pressure rose more than 20% above the baseline value obtained just before surgery commenced, which was predefined as a sign of experiencing pain. After completion of surgery, patients were extubated when adequate muscle strength was established, and they were transferred to the recovery room. Local anesthesia was not applied to wounds.

The postoperative pain management schedule was identical in both groups. In the recovery room, each patient received a PCA pump with nalbuphine (1 mg/mL^−1^), and the device was programmed to allow a self-administered bolus dose of 1 mg nalbuphine with a lockout time of 5 min. Additionally, patients were given 1 g intravenous paracetamol every 6 h. If the VAS exceeded 40 mm on any measurement, in addition, dexketoprofen 50 mg (with 8 h intervals) was given intravenously. All medications except nalbuphine were administered by an anesthesiologist nurse.

In the ESPB group, blocks were performed under sedoanalgesia before general anesthesia induction in the operating room. Following routine monitoring and premedication, the patients were placed in the sitting position. ESPB was performed under ultrasonographic guidance. The linear ultrasound transducer was placed in a longitudinal parasagittal orientation 3 cm lateral to the T7 spinous process. The erector spinae muscles were identified superficial to the tip of the T7 transverse process. The patient’s skin was anesthetized with 3 mL of 2% lidocaine subcutaneously. The tip of the 22 G needle was placed into the fascial plane on the deep (anterior) aspect of erector spinae muscle. The location of the needle tip was confirmed by visible fluid spread lifting the erector spinae muscle off the bony shadow of the transverse process on ultrasonographic imaging of the transverse process. A total of 20 mL of 0.5% bupivacaine was injected to this site. In addition, only in ESPB group, 0.1 mg/kg dexamethasone was administered intravenously. This block was performed by an anesthesiologist.

All patients qualified for PCNL had contrast-enhanced computed tomography performed before surgery. Briefly patients were operated in the supine position with the use of Amplatz dilators and sheaths. Patients were operated under general anesthesia. Perioperatively, 1.5 g cephalosporin (Cefuroxime) 2nd generation was used intravenously as antibiotic prophylaxis. Puncture and tract formation were performed by a urologist under fluoroscopic and ultrasonographic guidance. The formation of the tract was completed by insertion of a 30 Fr Amplatz sheath (Cook Medical). We used a 26 Fr rigid nephroscope (Storz) with ultrasound and a pneumatic lithotripter to disintegrate the stone. In case of an inaccessible (for rigid instrument) stone, we used a flexible optical cystonephroscope (CYF-5, Olympus) to retrieve the stone with a basket or disintegrated it with a holmium laser. At the end of the procedure, a re-entry Malecot (16 Fr) nephrostomy was inserted and maintained for 2 days. The procedure lasted approximately 90–120 min.

### 2.7. Statistical Analysis

All the continuous variables were checked for normality with the Kolmogorov–Smirnov test and for homogeneity of variance with Levene’s test. For variables with normal distribution, t-tests were applied. For continuous variables without normal distribution, the Mann–Whitney U-test was used. Statistics for categorical variables were computed with the chi-square test with Yates’s correction. *p* values < 5% were considered significant. Pearson correlation coefficients between continuous variables were computed. To assess the changes in VAS and MAP parameters, a multivariate general linear model was used (Hotelling’s test). To define the differences between subgroups, we used contrast analysis (planned comparisons). Variable VAS had lognormal distribution; thus, logarithmic transformation was performed before further analyses. After final analysis, the data were transformed backward to show the results in tables and graphs on an appropriate scale. Statistica 13 (Statsoft) was used for statistical analyses.

## 3. Results

**Figure 1:**
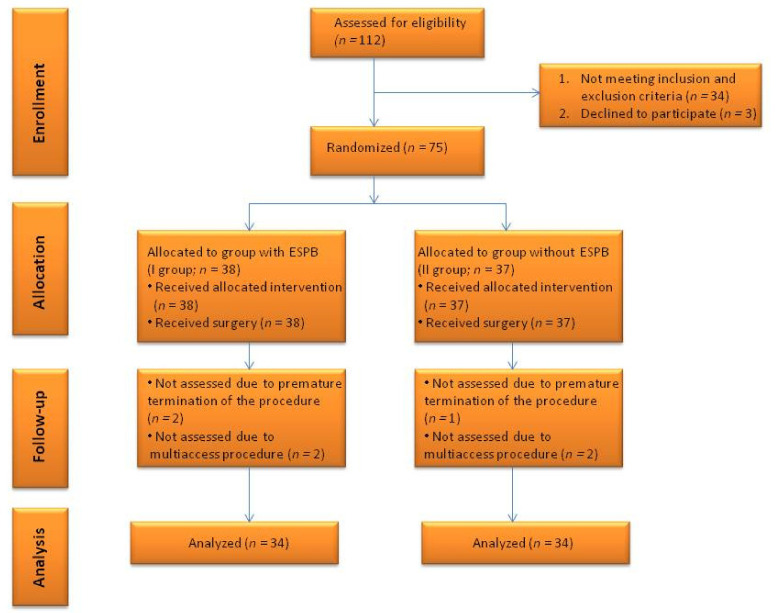
Consolidated Standards of Reporting Trials: flowchart for the trial.

Between January 2019 and May 2020, 112 patients with kidney stone disease were referred to our department to perform PCNL, 75 of whom were eligible for participation in the study and provided their informed consent. They were randomized to one of two groups. Randomization was performed with closed opaque envelopes with 1:1 allocation. The experimental group (group 1) consisted of patients who received ESPB right before general anesthesia and the control group (group 2) was without ESPB. After the surgery, seven patients were disqualified from the study. Finally, the first and the second group consisted of 34 patients for whom data were analyzed (Figure 1).

The demographic and perioperative characteristics are provided in Table 1 and Table 2. As shown, the potential confounding variables were evenly distributed between the groups. The preliminary analysis revealed a significant and negative linear correlation between VAS (both 1 and 2 h postoperatively) and BMI (r = −0.40, *p* = 0.001; and r = −0.32, *p* = 0.007, respectively). This correlation was also highly significant in each group separately (with and without ESPB; *p* < 0.01). There was no significant correlation between BMI and VAS assessed 4, 6, 12, and 24 h postoperatively.

As expected, we found a significant and positive correlation between average VAS and nalbuphine consumption (r = 0.42, *p* < 0.001).

**Figure 2:**
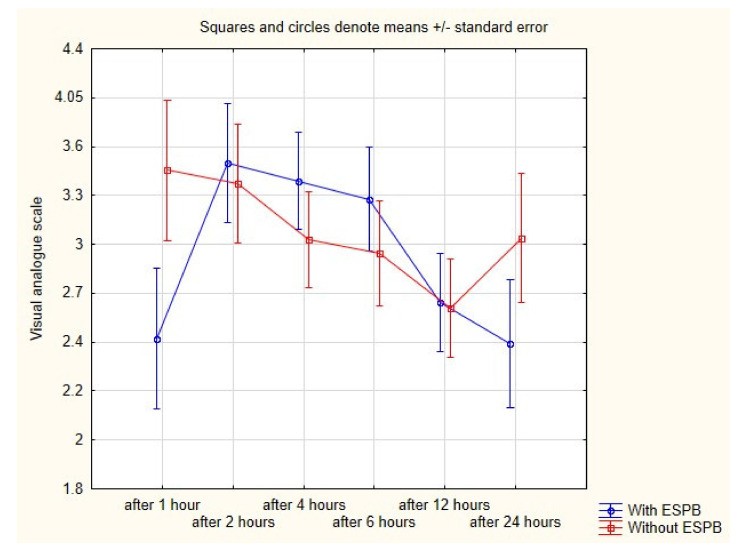
Visual analogue scale after operation in both groups.

The average VAS from all measurements was almost the same in groups 1 and 2 (Table 3). However, we found that VAS after one hour postoperatively was significantly lower in patients who received ESPB (2.3 vs. 3.3; *p* = 0.01). Other VAS measurements were not different between groups (Figure 2).

To identify factors influencing VAS one hour postoperative, we used stepwise regression with backward elimination. We found that the model with ESPB (*p* = 0.001), BMI (*p* = 0.004), nalbuphine consumption (*p* = 0.001), and accesses calyx (upper vs. lower; *p* = 0.02) had the highest and significant (*p* < 0.001) predictive value (adjusted R^2^).

We did not find any differences between analgesic consumption in PCA pump between groups or the need for rescue analgesia (Table 3).

**Figure 3:**
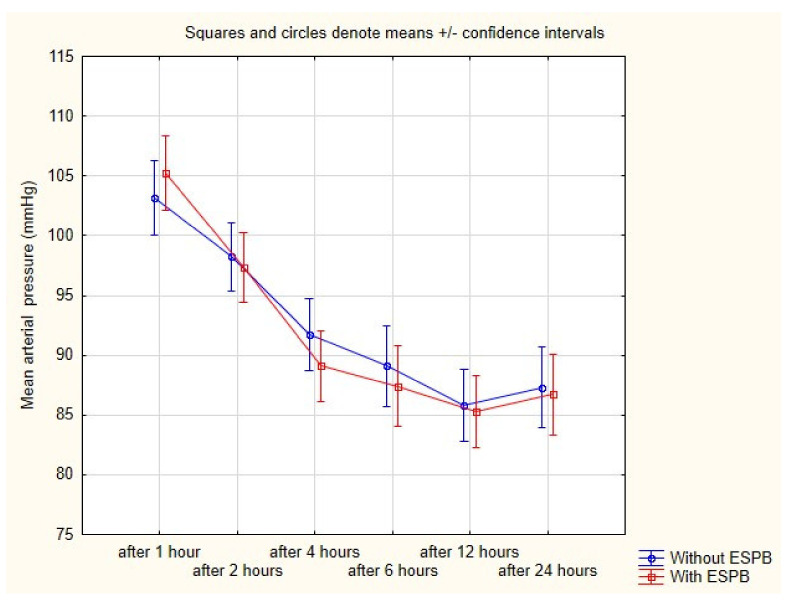
Mean arterial pressure after operation in both groups.

Safety measures were insignificantly different between groups. MAP was very similar in both groups during the 24 h postoperative period (Figure 3). Likewise, the rate of nausea and vomiting was comparable between groups. The median Ramsey scale from all measurements was two (Table 3). We did not observe any complications after ESPB such as pneumothorax or artery puncture.

## 4. Discussion

We examined the effectiveness of ESPB for postoperative pain treatment after PCNL. In this novel study, we found that such treatment has the potential to become widely used. Unfortunately, ESPB with bupivacaine was effective only in a very short postoperative period, but it shows potential for future research. Upcoming trials may test the effectiveness of long-lasting drugs in ESPB or the application of other drugs with the ability to prolong the action of bupivacaine.

PCNL is a highly efficacious procedure with stone-free rates exceeding 80% [9,10]. To date, it is considered the most effective treatment for large kidney stones, but drawbacks include higher blood loss, pain, and the complication rate in comparison with other forms of endourological treatment. Young age and male sex are associated with higher narcotic use postoperatively; however, we did not find such a relationship in our study [11]. Contrary to others, we found that upper pole access in the model strengthened its predictive value (adjusted R^2^) [12]. Interestingly, BMI had a significant negative impact on VAS assessed 1 and 2 h postoperatively, but there was no correlation in VAS assessed after 2 h. Our assumption is that patients with greater BMI have more fat tissue around the tract, which limits the spread of hematoma around the kidney after PCNL. Such hematoma in thinner patients may exert higher pressure on the kidney capsule and cause higher postoperative pain. It is possible that nociceptors adapt to higher extrarenal pressure shortly after operation and then the perceived pain originates only from the wound. However, this hypothesis requires confirmation in another study.

There are multiple methods to diminishing the intensity of postoperative pain. It may be lowered after tubeless and totally tubeless procedures [13,14] or applying a small-bore nephrostomy tube [15]. Scientific findings support using paravertebral block or epidural anesthesia before PCNL to minimize the postoperative pain [16,17]. Local injection of analgesic drugs is also effective for this treatment [18,19,20,21]. Preventive preoperative intravenous or subarachnoid spinal analgesia was also described [22,23].

Epidural anesthesia and paravertebral blocks are considered effective pain treatment that lowers postoperative opioid consumption and the rate of complications caused by these drugs [24,25]. However, they require well-trained medical personnel as there is a risk of significant complications [26,27]. To maintain the benefits of regional anesthesia while diminishing the possible complications, fascial plane blocks were evaluated and proved effective. The pioneer studies showed its efficacy after ventral hernia or mastectomy [1,5]. It is regarded a safe procedure; however, complications such as pneumothorax or artery puncture rarely occur [28].

To date, there were no studies regarding this kind of postoperative analgesia after PCNL. We showed that ESPB is an effective treatment of pain but limited to a very short postoperative period, which is the disadvantage in comparison with paravertebral block or epidural anesthesia. However, the simplicity of performing ESPB and the virtual lack of complications after this procedure supports its wide used before PCNLs. It is also possible that due to mobilization of patients one hour after surgery, they perceive higher pain after this specific time point.

The question arises as to why the nalbuphine consumption was almost the same in both groups since ESPB lowers the pain in the first postoperative hour. As shown in Figure 2, the pain was most intense in first postoperative hours. In the direct postoperative period, patients use the maximal acceptable dose in PCA irrespective of whether they received ESPB or not. Even if they used the maximal nalbuphine dose in PCA, they perceived lower pain because of ESPB.

Our study has its limitations. Firstly, the study was conducted in a single tertiary care center. Secondly, random anesthesiologists with different experience in this kind of treatment conducted the ESPB. However, all of them performed the procedure routinely. Thirdly, the study lacks the true double-blind design. The control group did not receive dexamethasone intravenously which could have impacted the final results. Another limitation is the lack of comparison between the groups in terms of the total amount of remifentanil administered during surgery. This comparison could reveal significant differences between the groups.

## 5. Conclusions

ESPB is an effective pain treatment after PCNL, but its effectiveness is limited to a very short postoperative period.

## Figures and Tables

**Table 1:** Demographic characteristics of the analyzed groups. BMI, body mass index; PCNL, percutaneous nephrolithotomy.

		ESPB Group (*n* = 34)	Control Group (*n* = 34)	*p* Value
Age, years, mean (SD)		55.2 (10)	57.4 (11,3)	0.41
Sex, no. (%)				
	Men	15 (44.1)	22 (64.7)	0.14
	Women	19 (55.8)	12 (35.2)	
Diabetes, no. (%)				
	Yes	2 (5.8)	7 (20.5)	0.15
	No	32 (94.1)	27 (79.4)	
Hypertension, no. (%)				
	Yes	10 (29.4)	15 (44.1)	0.31
	No	24 (70.5)	19 (55.8)	
BMI, kg/m^2^, mean (SD)		28.5 (3.6)	28.7 (3.7)	0.81
First PCNL, no. (%)				
	Yes	30 (88.2)	25 (73.5)	0.21
	No	4 (11.7)	9 (26.4)	

**Table 2:** Perioperative characteristics of the analyzed groups.

		ESPB Group (*n* = 34)	Control Group (*n* = 34)	*p* Value
Stone diameter, mm (SD)		24.8 (9.8)	23.9 (9.3)	0.69
Site, no. (%)				
	Left	20 (58.8)	17 (50)	0.62
	Right	14 (41.1)	17 (50)	
Stone position, no. (%)				
	Calyx	9 (26.4)	12 (35.2)	0.59
	Pelvis	16 (47)	12 (35.2)	
	Staghorn	9 (26.4)	10 (29.4)	
Operation time, min. (SD)		89.9 (29.3)	88.2 (25.2)	0.79
Access, no. (%)				
	Subcostal	32 (94)	30 (88.2)	0.66
	Intercostal	2 (5.8)	4 (11.7)	
Access [calyx], no. (%)				
	Upper	2 (5.8)	4 (11.7)	0.1
	Middle	11 (32.3)	4 (11.7)	
	Lower	21 (61.7)	26 (76.4)	

**Table 3:** Postoperative characteristics of the analyzed groups. VAS, visual analogue scale; MAP, mean arterial pressure.

		ESPB Group (*n* = 34)	Control Group (*n* = 34)	*p* Value
Average VAS, cm (SD)		2.9 (1.3)	3 (1.3)	0.65
Nalbuphine consumption, mL (SD)		46 (12.8)	47.2 (13.2)	0.69
Average MAP, mmHg (SD)		91.8 (6.16)	92.5 (6.12)	0.63
Median Ramsey scale, points, median		2	2	0.51
Additional dexketoprofenum, *n* (%)				
	Yes	10 (29.4)	9 (26.4)	1
	No	24 (70.5)	25 (73.5)	
Nausea/vomiting, *n* (%)				
	Yes	6 (17.6)	5 (14.7)	1
	No	28 (82.3)	29 (85.2)	

## Data Availability

The data are stored in the Primary Investigator’s database.

## References

[1] Chin K.J., Adhikary S., Sarwani N., Forero M. (2017). The analgesic efficacy of pre-operative bilateral erector spinae plane (ESP) blocks in patients having ventral hernia repair. Anaesthesia.

[2] Petsas D., Pogiatzi V., Galatidis T., Drogouti M., Sofianou I., Michail A., Chatzis I., Donas G. (2018). Erector spinae plane block for postoperative analgesia in laparoscopic cholecystectomy: A case report. J. Pain Res..

[3] Das Adhikary S., Pruett A., Forero M., Thiruvenkatarajan V. (2018). Erector spinae plane block as an alternative to epidural analgesia for post-operative analgesia following video-assisted thoracoscopic surgery: A case study and a literature review on the spread of local anesthetic in the erector spinae plane. Indian J. Anaesth..

[4] Ueshima H., Otake H. (2017). Clinical experiences of ultrasound-guided erector spinae plane block for thoracic vertebra surgery. J. Clin. Anesth..

[5] Bashandy G.M.N., Abbas D.N. (2015). Pectoral nerves I and II blocks in multimodal analgesia for breast cancer surgery: A randomized clinical trial. Reg. Anesth. Pain Med..

[6] Ueshima H., Hiroshi O. (2018). Spread of local anesthetic solution in the erector spinae plane block. J. Clin. Anesth..

[7] Forero M., Adhikary S.D., Lopez H., Tsui C., Chin K.J. (2016). The erector spinae plane block a novel analgesic technique in thoracic neuropathic pain. Reg. Anesth. Pain Med..

[8] Tulgar S., Kapakli M.S., Senturk O., Selvi O., Serifsoy T.E., Ozer Z. (2018). Evaluation of ultrasound-guided erector spinae plane block for postoperative analgesia in laparoscopic cholecystectomy: A prospective, randomized, controlled clinical trial. J. Clin. Anesth..

[9] Bryniarski P., Paradysz A., Zyczkowski M., Kupilas A., Nowakowski K., Bogacki R. (2012). A randomized controlled study to analyze the safety and efficacy of percutaneous nephrolithotripsy and retrograde intrarenal surgery in the management of renal stones more than 2 cm in diameter. J. Endourol..

[10] Kim C.H., Chung D.Y., Rha K.H., Lee J.Y., Lee S.H. (2020). Effectiveness of Percutaneous Nephrolithotomy, Retrograde Intrarenal Surgery, and Extracorporeal Shock Wave Lithotripsy for Treatment of Renal Stones: A Systematic Review and Meta-Analysis. Medicina.

[11] Khater N., Keheila M., Lightfoot M., Shen J., Abourbih S., Alsyouf M., Li R., Baldwin D.D. (2017). Predictors of narcotic use after percutaneous nephrolithotomy. Can J. Urol..

[12] Lightfoot M., Ng C., Engebretsen S., Wallner C., Huang G., Li R., Alsyouf M., Olgin G., Smith J.C., Baldwin D.D. (2014). Analgesic use and complications following upper pole access for percutaneous nephrolithotomy. J. Endourol..

[13] Chen Z.-J., Yan Y.-J., Zhou J.-J., Zhou J.-J. (2020). Comparison of tubeless percutaneous nephrolithotomy and standard percutaneous nephrolithotomy for kidney stones: A meta-analysis of randomized trials ScienceDirect. Asian J. Surg..

[14] Moosanejad N., Firouzian A., Hashemi S.A., Bahari M., Fazli M. (2016). Comparison of totally tubeless percutaneous nephrolithotomy and standard percutaneous nephrolithotomy for kidney stones: A randomized, clinical trial. Braz. J. Med. Biol. Res..

[15] Pietrow P.K., Auge B.K., Lallas C.D., Santa-Cruz R.W., Newman G.E., Albala D.M., Preminger G.M. (2003). Pain after percutaneous nephrolithotomy: Impact of nephrostomy tube size. J. Endourol..

[16] Hatipoglu Z., Gulec E., Turktan M., Izol V., Arıdogan A., Gunes Y., Ozcengiz D. (2018). Comparative study of ultrasound-guided paravertebral block versus intravenous tramadol for postoperative pain control in percutaneous nephrolithotomy. BMC Anesthesiol..

[17] Li C., Song C., Wang W., Song C., Kong X. (2016). Thoracic Paravertebral Block versus Epidural Anesthesia Combined with Moderate Sedation for Percutaneous Nephrolithotomy. Med. Princ. Pract..

[18] Özdilek A., Beyoğlu Ç.A., Demirdağ Ç., Şen Ö., Erbabacan Ş.E., Ekici B., Altindaş F., Köksal G.M. (2020). Perioperative analgesic effects of preemptive ultrasound-guided subcostal transversus abdominis plane (TAP) block for percutaneous nephrolithotomy: A prospective, randomized trial. J. Endourol..

[19] Parikh G.P., Shah V.R., Vora K.S., Parikh B.K., Modi M.P., Kumari P. (2014). Ultrasound guided peritubal infiltration of 0.25% Bupivacaine versus 0.25% Ropivacaine for postoperative pain relief after percutaneous nephrolithotomy: A prospective double blind randomized study. Indian J. Anaesth..

[20] Kirac M., Tepeler A., Bozkurt O.F., Elbir F., Ozluk C., Armagan A., Unsal A., Biri H. (2013). The Efficacy of Bupivacaine Infiltration on the Nephrostomy Tract in Tubeless and Standard Percutaneous Nephrolithotomy: A Prospective, Randomized, Multicenter Study. Urology.

[21] Sharifi S.H.H., Soltani M.H., Rezaeetalab G.H., Sharif R.Y., Khaledi F., Lashay A., Sharifiaghdas F. (2014). Intermittent Perirenal Instillation of Bupivacaine After Tubeless Percutaneous Nephrolithotomy Under Spinal Anesthesia: A Double-Blind, Placebo-Controlled Clinical Trial. J. Endourol..

[22] Kaygusuz K., Gokce G., Ozdemir Kol I., Ayan S., Gursoy S. (2007). Efficacy of Preventive Analgesia with Tramadol or Lornoxicam for Percutaneous Nephrolithotomy: A Prospective, Randomized, Double-Blind, Placebo-Controlled Study. Curr. Ther. Res..

[23] Andreoni C., Olweny E.O., Portis A.J., Sundaram C.P., Monk T., Clayman R.V. (2002). Effect of Single-Dose Subarachnoid Spinal Anesthesia on Pain and Recovery after Unilateral Percutaneous Nephrolithotomy. J. Endourol..

[24] Wu C.L., Cohen S.R., Richman J.M., Rowlingson A.J., Courpas G.E., Cheung K., Lin E.E., Liu S.S. (2005). Efficacy of postoperative patient-controlled and continuous infusion epidural analgesia versus intravenous patient-controlled analgesia with opioids: A meta-analysis. Anesthesiology.

[25] El-Boghdadly K., Madjdpour C., Chin K.J. (2016). Thoracic paravertebral blocks in abdominal surgery-A systematic review of randomized controlled trials. Br. J. Anaesth..

[26] Halabi W.J., Kang C.Y., Nguyen V.Q., Carmichael J.C., Mills S., Stamos M.J., Pigazzi A. (2014). Epidural analgesia in laparoscopic colorectal surgery: A Nationwide analysis of use and outcomes. JAMA Surg..

[27] McCartney C.J.L., Mariano E.R. (2016). Education in Ultrasound-Guided Regional Anesthesia: Lots of Learning Left to Do. Reg. Anesth. Pain Med..

[28] Ueshima H. (2018). Pneumothorax after the erector spinae plane block. J. Clin. Anesth..

